# Impact on the Australian Quitline of new graphic cigarette pack warnings including the Quitline number

**DOI:** 10.1136/tc.2008.028290

**Published:** 2009-02-11

**Authors:** C L Miller, D J Hill, P G Quester, J E Hiller

**Affiliations:** 1 The Cancer Council South Australia, Eastwood, South Australia, Australia; 2 Discipline of Public Health, School of Population Health and Clinical Practice, University of Adelaide, South Australia, Australia; 3 The Cancer Council Victoria, Victoria, Australia; 4 Business School, University of Adelaide, South Australia, Australia

## Abstract

**Background:**

In March 2006, Australia introduced graphic pictorial warnings on cigarette packets. For the first time, packs include the Quitline number.

**Objective:**

To measure the combined effect of graphic cigarette pack warnings and printing the Quitline number on packs on calls to the Australian Quitline service.

**Methods:**

Calls to the Australian Quitline were monitored over 4 years, 2 years before and after the new packets were introduced.

**Results:**

There were twice as many calls to the Quitline in 2006 (the year of introduction), as there were in each of the preceding 2 years. The observed increase in calls exceeds that explained by the accompanying television advertising alone. While call volume tapered back in 2007, it remained at a level higher than before the introduction of new packets. No change was observed in the proportion of first time callers.

**Conclusion:**

Introducing graphic cigarette packet warnings and the Quitline number on cigarette packets boosts demand for Quitline services, with likely flow on effects to cessation.

In March 2006, graphic health warnings were included on cigarette and other tobacco packs in Australia. In addition, and for the first time, the Australian Quitline number was printed on packets. Prior to 2006, Australia had text-based warnings. There was an infoline number printed in small text on the side of the pack. This number diverted to the Quitline.

Like the text-based warnings that preceded them, the graphic health warnings are mandated under Australia’s Trade Practices Act,^1^ which includes regulations to inform and protect consumers. Graphic images and explanatory messages cover 30% of the front and 90% of the back of the pack. The message “You CAN quit smoking. Call the Quitline 131 848, talk to your doctor or pharmacist, or visit www.quitnow.info.au” is also included on the back of all packs. The Quitline number is also “stamped” on top of the graphic image on the backs of packs. Regulations prescribe the details of the size of the elements.^1^ There are 14 different warnings divided into 2 sets,^2^ ^3^ rotated semi-annually. Many but not all of the messages and images were new to Australian smokers. Currently, there is no provision to update the messages or images on packets that were introduced to consumers in 2006.

A series of mass media campaign activities accompanied the introduction of the new cigarette packet warnings. The Australian Government screened an awareness raising campaign in February 2006.^4^ In addition, a collaboration of Australian state and territory-based non-government health agencies developed a campaign to reinforce the pack warnings and promote quitting. This quit campaign featured two television commercials (TVCs) linked directly to the new graphic cigarette packet warnings; “Amputation”,^5^ linked to the warning “Smoking causes peripheral vascular disease” and “Mouth Cancer”,^5^ linked to the warning “Smoking causes mouth and throat cancer”. “Amputation” first aired in May 2006 and “Mouth Cancer” first aired in July 2006.

Australia is not the first country to introduce a Quitline or smokers’ helpline number on cigarette packets. In 2002, a smoking cessation message and quit line number were included on Dutch cigarette packets, along with prominent text warnings. This led to a 3.5-fold increase in calls to the Dutch Quitline.^6^ In the UK, written pack warnings, accompanied by a smoking helpline number, were reported as the second largest driver of callers to the UK National Health Service Stop Smoking Helpline.^7^ However, to date, no data have been published on the impact of the graphic cigarette packet warnings, accompanied by a Quitline number, on demand for a Quitline service.

It is well established that television advertising to promote quitting can increase calls to Quitlines^8^^–^10 and, therefore, quitting itself.^11^ This study measures the impact of new style cigarette packets, which included graphic cigarette packet warnings and the Quitline number, on calls to the Australian Quitline, and the extent to which call volume exceeded that which would be expected from the usual mass media cessation advertising.

## METHODS

### Quitline call data

The Australian Quitline can be accessed from anywhere in Australia by dialling 131848 or 13 QUIT (137848) for the price of a local call. The Telstra Analyser (Telestra, Melbourne, Australia), software of the telecommunications provider, provides data on volume of calls, call source (broken down by state and region), time and duration of calls.

Individual states and territories have their own databases of caller details. These data were examined in one jurisdiction (South Australia), where callers who spoke to a counsellor (51% of all callers) were asked routinely whether they had called the Quitline before.

### Advertising data

Television anti-smoking advertising is quantified using target audience rating points (TARPs), provided by a media agency ACNielsen (Sydney, Australia). TARPs are a standard measure of television advertising weight. TARPs are used to indicate the number of people within a certain demographic group that were exposed to an advertisement within a given period of time. For example, 100 TARPs for 1 week is equal to an average of 1 exposure per person in the target population within that week of the campaign. In the present study, the TARPs relate to the target audience of Australians aged ⩾18 years.

### Analyses

Data analyses were conducted with SPSS V. 15 (SPSS, Chicago, Illinois, USA). Linear regression analyses were used to estimate the effect on calls to the Quitline of television advertising and the introduction of graphic pack warnings using data from January 2004 to December 2007 inclusive. In regression modelling, calls to the Quitline were the dependent variable, TARPs were a continuous independent variable and separate dummy variables were created for 2006 and 2007. Although data were not distributed normally, data were not transformed as this did nothing to strengthen the resulting model.

## RESULTS

**Figure 1:**
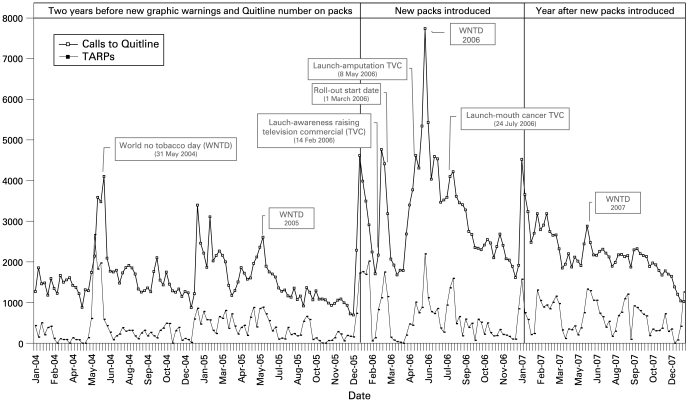
Calls to the Australian Quitline prior to and after the introduction of graphic cigarette packet warnings.

Figure 1 shows the volume of calls to the Australian Quitline service over a 4-year period. Every year, calls to the Quitline peak at New Year, around World No Tobacco Day (31 May) and coinciding with other major cessation campaigns. In 2006, the Australian Quitline received 164 850 calls. This compares with 81 490 calls in 2004, 84 442 calls in 2005 and 117 544 calls in 2007. The number of calls received in 2006, the year that new graphic cigarette packet warnings including the Quitline number were introduced, represents a doubling of calls received in either of the preceding 2 years. The number of calls received in 2006 was 40% higher than those received in 2007, the year after the warnings were introduced.

Calls increased markedly when new cigarette packet warnings were first introduced. Call volume levelled off in the weeks following the initial launch but built up again in subsequent months when the accompanying quit campaign TVCs were launched.

The linear regression model showed significant relationships between the independent variables and the dependent variable overall and had good overall explanatory value (F = 133.4; p<0.001; adjusted R^2^ = 0.657). The model predicted a base number of calls (constant B = 1161; t = 17.0, p<0.001); a significant linear relationship between every 100 TARPs and calls to the Quitline (B = 119.0; t = 12.6; p<0.001); and separate independent increases in calls were observed for years 2006 (B = 1236.2; t = 11.7; p<0.001); and 2007 (B = 341.0; t = 3.2; p = 0.001), above what was explained by TARPs alone. Call volume was still elevated in 2007, compared to 2004 and 2005, although there was erosion in call volume from 2006.

When a South Australia subsample of callers to the Quitline was examined further, it revealed that there was no increase in the proportion of first time callers in 2006 (77%), the year in which new pack warnings including the Quitline number were introduced, compared to 2005 (78%).

## DISCUSSION

Australia is a “mature” tobacco control market where most forms of tobacco promotion are banned, increasing the significance of the packet as a medium for marketing.^12^ The introduction of graphic health warnings on cigarette packets represented a major change in Australia. The new warnings are larger than the old text-based warnings, they are in colour, many feature confronting images known to have a strong impact on smokers (unpublished results) and, for the first time, they feature the Quitline number prominently. Graphic cigarette packet warnings provided a chance to communicate new information to Australian smokers in a new way. They went some way towards countering the glamourisation and promotion of tobacco through packet appearance.

**What this paper adds**

- Many countries are moving to introduce graphic cigarette packet warnings; some with a Quitline or helpline number.
- However, the impact on calls to the Quitline of graphic (in contrast to text-only) warnings with accompanying Quitline number has not previously been quantified.
- This study shows that even in a “mature” tobacco control environment such as Australia, such an intervention has considerable positive impact on demand for a Quitline, with positive implications for quitting.

Since the 1980s, most Australian state and territories have established strong anti-tobacco (quit) mass media campaigns, supported by the Australian Quitline. Because of the clear relationships between high-quality mass media campaigns, calls to the Quitline and quitting behaviour,^9^ ^11^ the introduction of graphic cigarette packet warnings was viewed by health agencies as an opportunity to reinforce and sustain any impact with tailored new mass media quit campaigns. As a consequence, it is not possible to completely separate the independent effects of the packs themselves and the accompanying mass media communications themed around the pack warnings.

However, the rise in calls to the Australian Quitline service observed in this study was substantial and sustained. The size and timing of the rise in calls, compared to the previous 2 years, indicates that this is highly likely to be due to the introduction of the new graphic cigarette packet warnings that included the Quitline number. The regression analysis also demonstrates that it is very unlikely that mass media alone explained the observed increase in calls because the introduction of the warnings had an independent effect. Further evidence that mass media quit campaigns were not the primary cause of increased calls is the fact that some of the increase in calls was observed prior to the launch of the quit campaigns. The Quitline number is a prominent but integrated component of the new-style warnings on Australian cigarette packets. There was no prominent display of the Quitline number on Australian cigarette packets prior to this, only the low-profile infoline number. Therefore it is not possible to separate the contributions of the components of the new warnings: namely the visual image, the large warning text, the detailed warning on the back of the packet or the Quitline number. Their impact has been measured as a whole.

There was no change in the proportion of first time callers, compared to the previous year, indicating that the intervention had a positive effect upon new quitters and repeat callers.

The observed increase in call volume did persist in the year following the introduction of the warnings (2007). Although there are 14 different warnings, with a scheduled rotation mechanism, it is likely that the reduction in call volume was due to a degree of “wear out”. This provides another example of a health promotion intervention having a positive effect more akin to a spring than a screw.^13^ The analogy is one about sustainability. Once driven down, a screw stays where it is whereas a spring needs ongoing pressure to avoid a rebound due to opposing force. Tobacco control initiatives, such as graphic warnings, compete in an environment with opposing forces, including below-the-line tobacco promotion and consumer adaptation levels to warnings. The apparent “wear out” of the initial impact of the warnings suggests the need for governments to be able to change warnings for the sake of maintaining novelty (and avoiding desensitisation) and to inform smokers of the hazards that come to light from research published since the set of warnings was prescribed.

In conclusion, the Australian Quitline experienced a doubling of calls upon introduction of graphic cigarette packet warnings that included a prominent Quitline number. Other countries with mature tobacco markets could expect a similar impact upon introduction of graphic warnings, especially if accompanied by reinforcing mass media activities. The flow-on effects in terms of quitting are likely to be substantial. Previous research has demonstrated that at 12 months, around 30% of callers to the Australian Quitline have succeeded in quitting smoking,^9^ making such warnings an important source of consumer information but also a worthwhile cessation intervention.
